# Functional Dissection of *HOXD* Cluster Genes in Regulation of Neuroblastoma Cell Proliferation and Differentiation

**DOI:** 10.1371/journal.pone.0040728

**Published:** 2012-08-07

**Authors:** Yunhong Zha, Emily Ding, Liqun Yang, Ling Mao, Xiangwei Wang, Brian A. McCarthy, Shuang Huang, Han-Fei Ding

**Affiliations:** 1 Cancer Center and Department of Pathology, Medical College of Georgia, Georgia Health Sciences University, Augusta, Georgia, United States of America; 2 Department of Biochemistry and Molecular Biology, Medical College of Georgia, Georgia Health Sciences University, Augusta, Georgia, United States of America; 3 Lakeside High School, Evans, Georgia, United States of America; 4 Department of Neurology, the First People's Hospital of Yichang, Three Gorges University College of Medicine, Yichang, Hubei, China; 5 Department of Neurology, Union Hospital, Tongji Medical College, Huazhong University of Science and Technology, Wuhan, Hubei, China; 6 Department of Urology, Second Affiliated Hospital, Third Military Medical University, Chongqing, China; University of Birmingham, United Kingdom

## Abstract

Retinoic acid (RA) can induce growth arrest and neuronal differentiation of neuroblastoma cells and has been used in clinic for treatment of neuroblastoma. It has been reported that RA induces the expression of several *HOXD* genes in human neuroblastoma cell lines, but their roles in RA action are largely unknown. The HOXD cluster contains nine genes (*HOXD1, HOXD3, HOXD4, and HOXD8-13*) that are positioned sequentially from 3′ to 5′, with *HOXD1* at the 3′ end and *HOXD13* the 5′ end. Here we show that all *HOXD* genes are induced by RA in the human neuroblastoma BE(2)-C cells, with the genes located at the 3′ end being activated generally earlier than those positioned more 5′ within the cluster. Individual induction of *HOXD8, HOXD9, HOXD10* or *HOXD12* is sufficient to induce both growth arrest and neuronal differentiation, which is associated with downregulation of cell cycle-promoting genes and upregulation of neuronal differentiation genes. However, induction of other *HOXD* genes either has no effect (*HOXD1*) or has partial effects (*HOXD3, HOXD4, HOXD11* and *HOXD13*) on BE(2)-C cell proliferation or differentiation. We further show that knockdown of HOXD8 expression, but not that of HOXD9 expression, significantly inhibits the differentiation-inducing activity of RA. HOXD8 directly activates the transcription of *HOXC9*, a key effector of RA action in neuroblastoma cells. These findings highlight the distinct functions of *HOXD* genes in RA induction of neuroblastoma cell differentiation.

## Introduction

Retinoic acid (RA), a derivative of vitamin A, has a key role in vertebrate morphogenesis, cellular differentiation, and tissue homeostasis. In embryonic development of the nervous system, RA is essential for the organization of the posterior hindbrain and anterior spinal cord, and for the differentiation and specification of motor neurons [1], [2]. This function of RA depends critically on its ability to regulate the expression of *HOX* genes that encode a family of transcriptional factors with the ability to specify positional identities of tissues along the anteroposterior axis [3], [4], [5]. In vitro, RA can induce neuronal differentiation of embryonal carcinoma stem cells [6], [7], embryonic stem cells [8] and neuroblastoma cells [9], which is associated with induction of *HOX* genes [10], [11], [12], [13], [14], [15]. However, the precise roles of these upregulated *HOX* genes in RA-induced neuronal differentiation are not well understood.

Neuroblastoma is a common childhood malignant tumor of the sympathetic nervous system that arises either in the adrenal medulla or in paravertebral sympathetic ganglia [16], [17]. It has long been recognized that neuroblastoma differentiation states strongly affect clinical outcomes: patients with neuroblastomas of differentiating histology have a significantly better chance of survival than those with undifferentiated or poorly differentiated neuroblastomas [18], [19], [20], [21], [22]. Because of its ability to induce differentiation of neuroblastoma cells, RA has been used in clinic as a therapeutic agent for high-risk neuroblastomas [23], [24]. Thus, identification of the downstream effectors of RA signaling and elucidation of the modes of their action may identify new drug targets for differentiation-based neuroblastoma therapy.

We have recently reported that high-level HOXC9 expression is associated with neuroblastoma differentiation and is prognostic for better survival in neuroblastoma patients. HOXC9 induces the growth arrest and neuronal differentiation of neuroblastoma cells by directly targeting both cell cycle-promoting and neuronal differentiation genes. HOXC9 expression is upregulated by RA and knockdown of HOXC9 expression confers resistance to RA-induced growth arrest and differentiation. These findings identify HOXC9 as a key regulator of neuroblastoma differentiation [25]. In addition to HOXC9, it has been reported that RA treatment of neuroblastoma cells leads to a significant induction of several *HOXD* genes [13], [14], [15]. In this report, we present a comprehensive analysis of RA induction of *HOXD* genes in the regulation of neuroblastoma cell proliferation and differentiation.

## Results

### RA induction of HOXD genes in neuroblastoma cells

We conducted this study in BE(2)-C cells, an RA-sensitive human neuroblastoma cell line enriched for cells capable of self-renewal and multi-lineage differentiation [26], [27]. BE(2)-C cells treated with RA display morphologic changes characteristic of neuronal differentiation and G1 arrest [25], [26], [27]. Previous studies with the human neuroblastoma cell lines SK-N-BE, CHP-134, SK-N-SH, LA-N-1 and LA-N-5 using standard RT-PCR have revealed marked induction of *HOXD1* and *HOXD8* following RA treatment in all of the neuroblastoma cell lines, as well as low levels of *HOXD4* and *HOXD9* induction in some neuroblastoma cell lines [14], [15].

**Figure 1:**
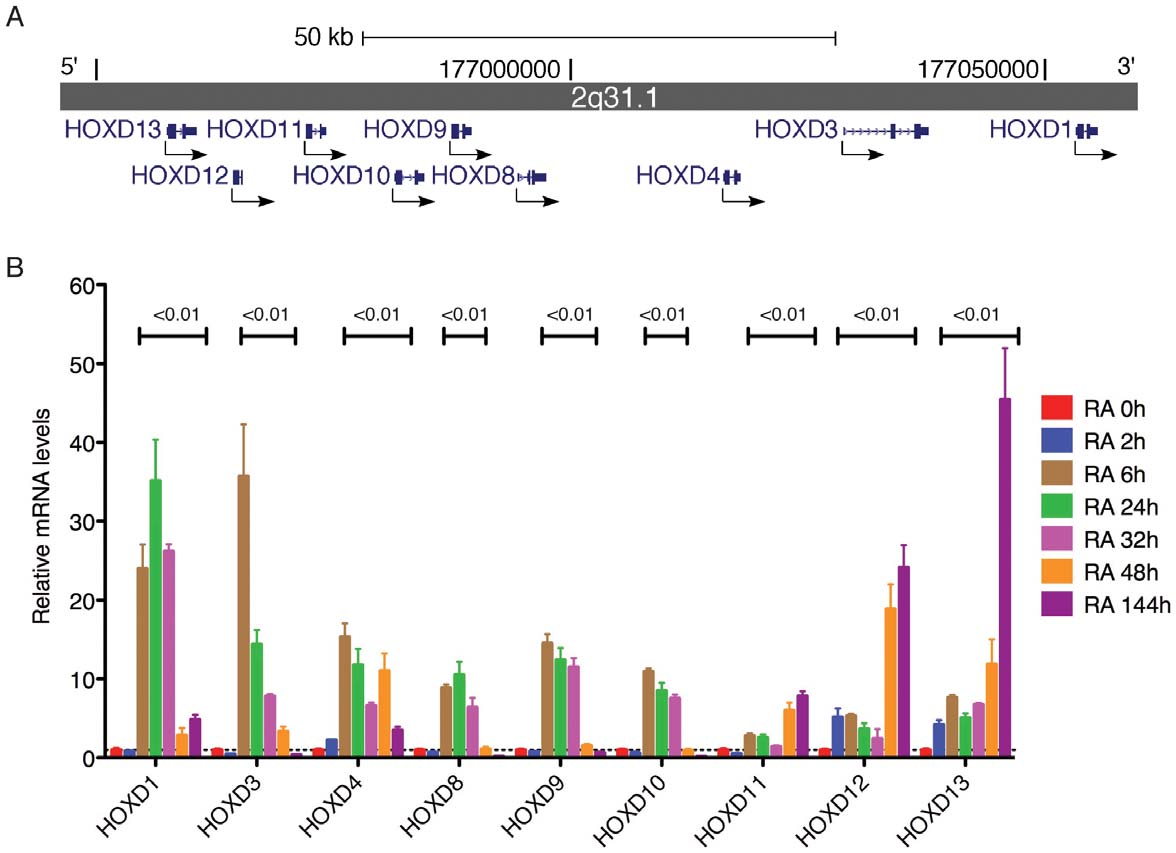
RA induction of *HOXD* genes in BE(2)-C cells. (**A**) Genomic structure of the human *HOXD* cluster on chromosome 2q31.1, adapted from the UCSC Genome Browser. Arrows indicate the transcriptional direction of *HOXD* genes. (**B**) qRT-PCR analysis of *HOXD* mRNA levels at various time points following RA (10 µM) treatment. The *HOXD* mRNA levels in BE(2)-C cells immediately before RA treatment (RA 0h) were designated as 1.0 (dashed line). The data were from two independent samples with each being assayed in triplicates and analyzed using two-tailed Student's *t*-test with the *p* values indicated. Error bars, standard deviation (SD).

The human *HOXD* complex contains nine genes *HOXD1, HOXD3, HOXD4, HOXD8, HOXD9, HOXD10, HOXD11, HOXD12* and *HOXD13*, which are clustered from 3′ to 5′ in an approximately 100-kb stretch on chromosome 2q31.1, with *HOXD1* at the 3' end and *HOXD13* the 5′. end (Figure 1 A). We performed two independent time-course studies of RA-induction of *HOXD* genes in BE(2)-C cells that were treated continuously with RA. Quantitative RT-PCR showed a marked increase (∼10- to 50-fold) in mRNA levels for all *HOXD* genes following RA treatment, relative to their levels in untreated cells (Figure 1 B). The transcriptional activation of *HOXD* genes by RA occurred in an apparent two-stage order colinear with their 3′ to 5′ arrangement in the cluster: marked induction (>10 fold) of the six 3′ *HOXD* genes (*HOXD1, HOXD3, HOXD4, HOXD8, HOXD9* and *HOXD10*) occurred essentially at the same time, as early as 6 h following RA treatment, whereas similar levels of induction (>10 fold) of *HOXD11, HOXD12* and *HOXD13* was not observed until 48 h after RA treatment (Figure 1 B). The transcriptional upregulation of the six 3′ *HOXD* genes was transient and their mRNA levels declined substantially by 48 h. By contrast, the mRNA levels of *HOXD11, HOXD12* and *HOXD13* continued to increase for at least 6 days (Figure 1 B). Overall, these findings are consistent with the phenomenon of “RA sensitivity colinearity” that was first observed in human embryonal carcinoma cells and later confirmed in the mouse embryo: *HOX* genes located at the 3′ end are activated generally earlier by RA than those positioned more 5′ within the cluster [28], [29]. These observations suggest that RA induction of *HOXD* genes in neuroblastoma cells is of physiological relevance.

### Distinct functions of HOXD proteins in regulation of neuroblastoma cell proliferation

To assess the functional consequence of *HOXD* induction by RA in neuroblastoma cells, we generated BE(2)-C-derived cell lines with inducible expression of individual *HOXD* genes in the absence of doxycycline. Quantitative RT-PCR analysis revealed that the mRNA levels of most *HOXD* genes were increased between 40- to 60-fold by day 3 following doxycycline removal (Figure S1 A). The lowest induction was observed with *HOXD9* (∼25-fold) and the highest with *HOXD13* (∼70-fold) (Figure S1 A). The induction of HOXD proteins was confirmed by immunoblot analysis using an antibody against the Myc-tag (Figure S1 B).

**Figure 2:**
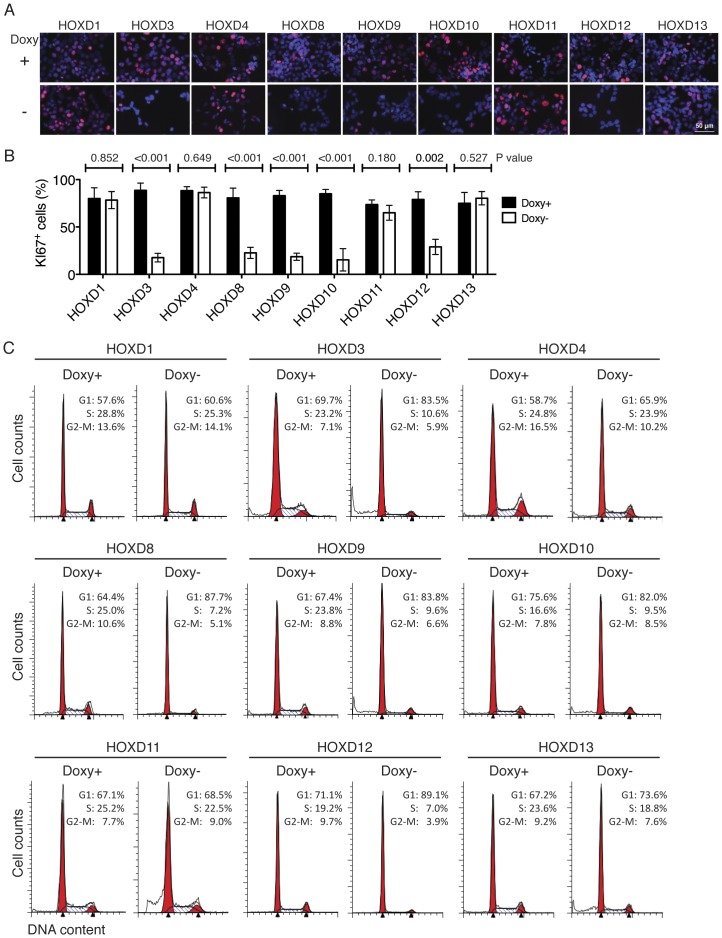
Distinct functions of HOXD proteins in regulation of cell proliferation. (**A–B**) Immunofluorescent staining (**A**) and quantification (**B**) of KI67-expressing BE(2)-C/Tet-Off/myc-HOXD cells cultured in the presence (Doxy+, 2 µg/ml) or absence doxycycline (Doxy-) for 6 days. Scale bars, 50 µm. The data in (**B**) were obtained from at least 5 randomly selected 400x fields (∼400 DAPI-positive cells) and analyzed using two-tailed Student's *t*-test with *p* values indicated. Error bars, SD. (**C**) FACS analysis of the cell cycle status of BE(2)-C/Tet-Off/myc-HOXD cells cultured in the presence or absence of Doxy for 6 days. Shown are representative of two independent experiments with similar results.

We first examined the effect of individual *HOXD* genes on the proliferation of BE(2)-C cells. Induction of *HOXD3, HOXD8, HOXD9, HOXD10* or *HOXD12* led to a marked decrease in the number of cells that expressed KI67 (Figure 2 A-B), a nuclear protein specifically expressed in proliferating cells [30]. By contrast, no such effect was observed following the induction of *HOXD1, HOXD4, HOXD11* or *HOXD13* (Figure 2 A-B). Consistent with the finding, fluorescence-activated cell sorting (FACS) showed that individual induction of *HOXD3, HOXD8, HOXD9, HOXD10* or *HOXD12* caused G1 arrest, whereas induction of *HOXD1, HOXD4, HOXD11* or *HOXD13* had no significant effect on cell cycle progression (Figure 2 C). Together, these findings demonstrate distinct functions of HOXD proteins in regulation of neuroblastoma cell proliferation, with only HOXD3, HOXD8, HOXD9, HOXD10 and HOXD12 proteins being able to recapitulate the G1 arrest phenotype induced by RA.

### Anti-growth HOXD proteins downregulate the expression of cell cycle-promoting genes

**Figure 3:**
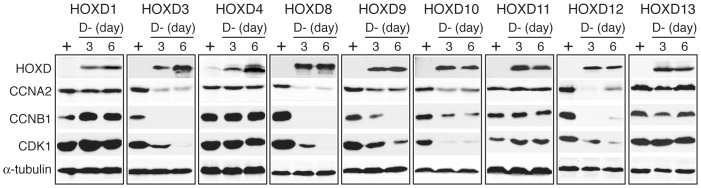
Distinct functions of HOXD proteins in regulation of cyclin and CDK expression. Immunoblot analysis of cyclin A2, cyclin B1 and CDK1 levels in BE(2)-C/Tet-Off/myc-HOXD cells cultured in the presence or absence of Doxy for 3 or 6 days. Alpha-tubulin levels are shown as loading control. Shown are representative of two independent experiments with similar results.

Cell cycle progression is driven by cyclins and cyclin-dependent kinases (CDKs) [31], [32]. RA-induced G1 arrest is associated with downregulation of cyclin A2, cyclin B1 and CDK1 [25], [33], [34]. This effect of RA could be fully recapitulated by individual induction of *HOXD3, HOXD8, HOXD9, HOXD10* or *HOXD12*, which all led to significant downregulation of cyclin A2, cyclin B1 and CDK1 (Figure 3). By contrast, the HOXD proteins that failed to induce G1 arrest (HOXD1, HOXD4, HOXD11 and HOXD13) had no apparent effects on the protein levels of cyclin A2, cyclin B1 and CDK1 (Figure 3).

We further examined the specificity of these anti-growth HOXD genes in downregulation of cyclins and CDKs. Induction of *HOXD3, HOXD8, HOXD9, HOXD10* or *HOXD12* had no significant effect on the expression levels of cyclin D1, CDK4 and CDK6, which are essential for maintaining cells in the G1 phase of the cell cycle (Figure S2). Together, these findings suggest that HOXD3, HOXD8, HOXD9, HOXD10 and HOXD12 inhibit cell proliferation by downregulating cyclins and CDKs that are required for cell cycle progression through the S, G2 and M phases.

### Distinct functions of HOXD proteins in regulation of neuroblastoma cell differentiation

**Figure 4:**
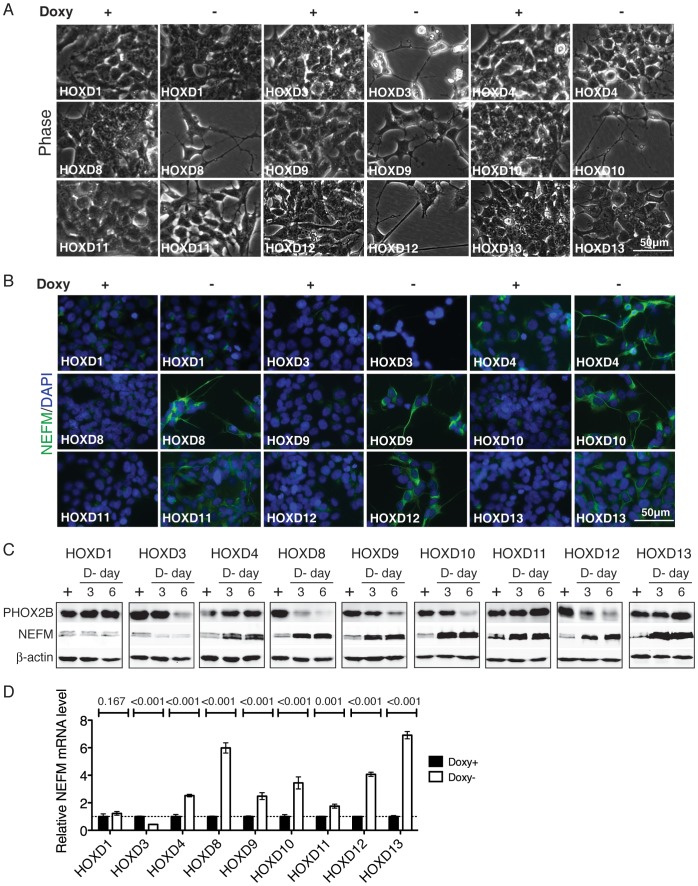
Distinct functions of HOXD proteins in regulation of neuronal differentiation. (**A**–**B**) Phase contrast imaging (**A**) and NEFM immunofluorescent staining (**B**) of BE(2)-C/Tet-Off/myc-HOXD cells cultured in the presence or absence of Doxy for 7 days. Scale bars, 50 µm. Shown are representative of three independent experiments with similar results. (**C**) Immunoblot analysis of PHOX2B and NEFM protein levels in the same cell samples described in Figure 3. Beta-actin levels are shown as loading control. Shown are representative of two independent experiments with similar results. (**D**) Quantification of *NEFM* mRNA levels in BE(2)-C/Tet-Off/myc-HOXD cells cultured in the presence or absence of Doxy for 3 days. The *NEFM* mRNA levels in BE(2)-C/Tet-Off/myc-HOXD cells in the presence of Doxy were designated as 1.0 (dashed line). The data were from two independent samples with each being assayed in triplicates and analyzed using two-tailed Student's *t*-test with the *p* values indicated. Error bars, SD.

BE(2)-C cells treated with RA display morphologic changes characteristic of neuronal differentiation with extensive outgrowth of neurites [25], [26], [27]. At the molecular level, RA-induced neuronal differentiation is characterized by downregulation of progenitor cell markers, for example, paired-like homeobox 2B (PHOX2B) [25], [35], and upregulation of neuronal differentiation markers, such as neurofilament medium polypeptide (NEFM) [25], [36]. Individual induction of *HOXD8, HOXD9, HOXD10* or *HOXD12* in BE(2)-C cells also led to morphologic changes of neuronal differentiation with marked neurite outgrowth (Figure 4 A). Consistent with the morphologic change, these cells displayed a significant reduction in PHOX2B expression and a marked increase in NEFM expression (Figure 4 B–D). These observations indicate that induction of *HOXD8, HOXD9, HOXD10* or *HOXD12* can recapitulate the neuronal differentiation phenotype induced by RA.

By contrast, induction of *HOXD1* in BE(2)-C cells resulted in no apparent changes in both morphology and expression levels of PHOX2B and NEFM (Figure 4). The remaining HOXD proteins varied in their ability to induce neuronal differentiation in BE(2)-C cell. HOXD3 was able to induce neurite outgrowth and downregulation of PHOX2B, but failed to upregulate NEFM expression (Figure 4). In fact, HOXD3 induction resulted in a marked downregulation of NEFM expression (Figure 4 B-D). On the other hand, induction of HOXD4, HOXD11 or HOXD13, while having no significant effect on morphologic differentiation and PHOX2B expression, was able to markedly upregulate NEFM expression (Figure 4). Taken together, these findings suggest distinct roles of HOXD proteins in the control of morphologic differentiation and neuronal marker expression in neuroblastoma cells.

### HOXD8 is a mediator of RA action

**Figure 5:**
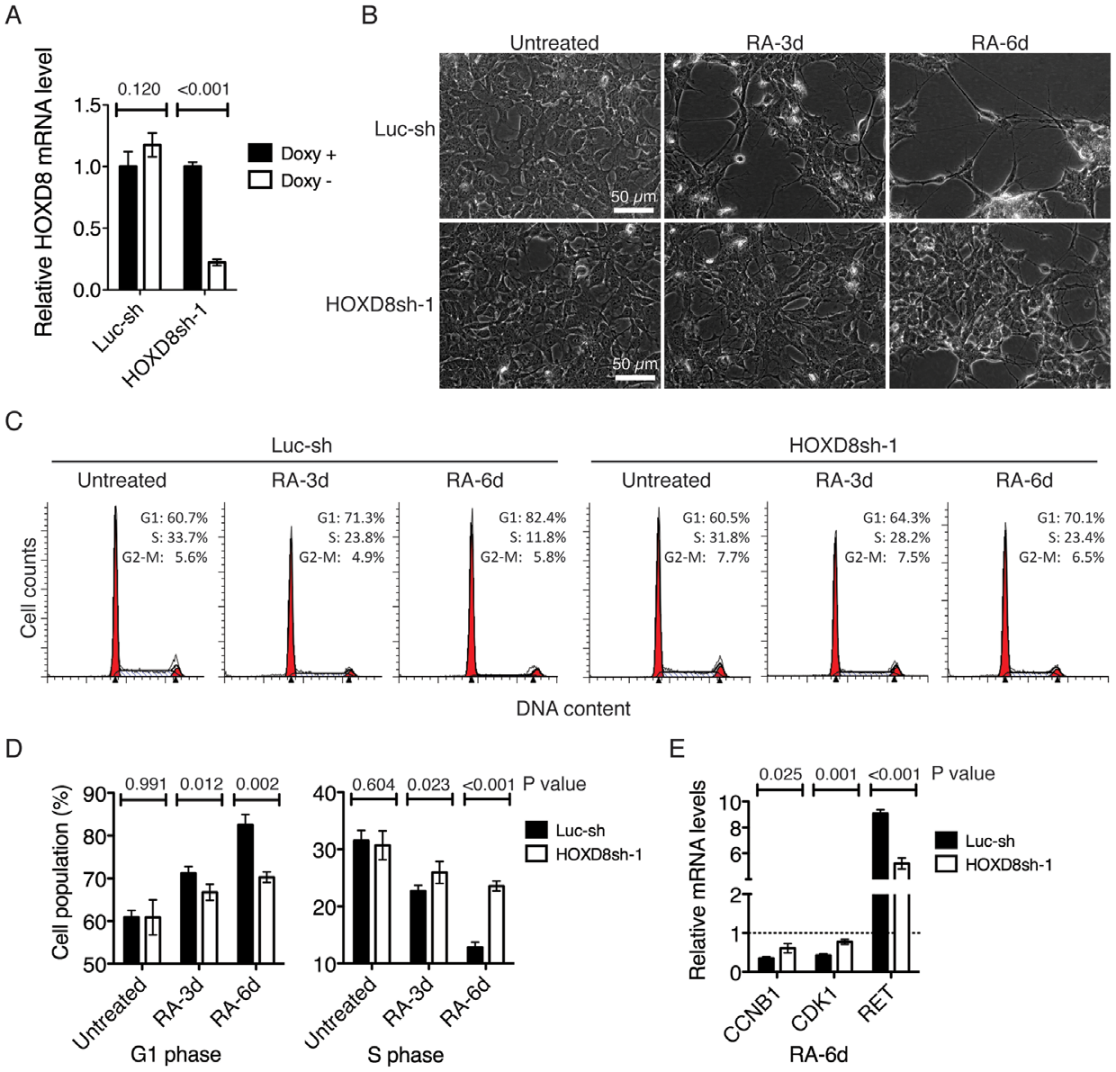
HOXD8 is a mediator of RA action. (**A**) qRT-PCR analysis of HOXD8 mRNA levels in BE(2)-C/Tet-Off cells with inducible expression of an shRNA sequence against Firefly *luciferase* (Luc-sh) or human *HOXD8* (HOXD8sh-1) in the absence of Doxy for 6 days. The *HOXD8* mRNA levels in BE(2)-C/Tet-Off/luc-sh cells in the presence of Doxy were designated as 1.0. The data were from two independent samples with each being assayed in triplicate and analyzed using two-tailed Student's *t*-test with the *p* values indicated. (**B–D**) Phase contrast imaging (**B**) and cell cycle analysis (**C–D**) of BE(2)-C/Tet-Off/Luc-sh and BE(2)-C/Tet-Off/HOXD8sh-1 cells that were cultured in the absence of Doxy for 6 days and then either untreated or treated with 10 µM RA for 3 or 6 days. The data in (**D**) were from three independent experiments and analyzed using two-tailed Student's *t*-test with the *p* values indicated. (**E**) qRT-PCR analysis of the mRNA levels of *CCNB1, CDK1,* and *RET* in BE(2)-C/Tet-Off/Luc-sh and BE(2)-C/Tet-Off/HOXD8sh-1 cells that were cultured in the absence of Doxy for 6 days and then treated with 10 µM RA for 6 days. The mRNA levels of the same genes in BE(2)-C/Tet-Off/Luc-sh cells cultured in the absence of Doxy without RA treatment were designated as 1.0 (dashed line). The data were from two independent samples with each being assayed in triplicate and analyzed using two-tailed Student's *t*-test with the *p* values indicated. Error bars (**A**, **D**, and **E**), SD.

Previous studies have revealed a marked induction of HOXD1 and HOXD8 following RA treatment in several human neuroblastoma cell lines examined including SK-N-BE, CHP-134, SK-N-SH, LA-N-1 and LA-N-5 [14], [15]. Since induction of HOXD8, but not of HOXD1, was able to recapitulate the phenotype of G1 arrest and neuronal differentiation induced by RA (Figures 2, 3, 4), we investigated the possibility of HOXD8 as a mediator of RA action. We generated two BE(2)-C-derived cell lines with inducible expression of short hairpin RNA (shRNA) sequences against different regions of the human *HOXD8* cDNA in the absence of doxycycline. Both sequences were highly effective in knockdown of HOXD8 expression, with HOXD8sh-1 reducing HOXD8 expression by 78% (Figure 5 A) and HOXD8sh-2 by 62% (Figure S3 A) relative to the control BE(2)-C cells with inducible expression of an shRNA sequence against the firefly *luciferase* gene (Luc-sh).

We examined the effect of HOXD8 knockdown on the ability of RA to induce G1 arrest and neuronal differentiation primarily with the BE(2)-C cells expressing HOXD8sh-1, which showed a more pronounced inhibition of HOXD8 expression compared to the cells expressing HOXD8sh-2 (Figure 5 A and Figure S3 A). BE(2)-C cells with HOXD8 knockdown were markedly resistant to RA-induced neuronal differentiation and G1 arrest, as determined by morphology (Figure 5 B) and cell cycle analysis (Figure 5 C-D). Consistent with these observations, knockdown of HOXD8 significantly abrogated the ability of RA to downregulate cell cycle-promoting genes and to upregulate RET, the receptor for glial cell-derived neurotrophic factor (Figure 5 E) and an RA-responsive gene [25]. We obtained similar results with the BE(2)-C cells expressing HOXD8sh-2, which also showed marked resistance to RA-induced G1 arrest (Figure S3 B). The finding that two different HOXD8 shRNA sequences had a similar effect on RA action provided evidence that the observed RA resistant phenotype resulted from HOXD8 knockdown. Together, these findings suggest that HOXD8 is a key mediator of the RA-induced differentiation program.

In addition, we investigated the role of HOXD9 in mediating RA action in BE(2)-C cells given the functional similarity between HOXD9 and HOXC9, which has recently been identified as a key effector of RA action in neuroblastoma cells [25]. We generated a BE(2)-C-derived cell line with inducible expression of an shRNA sequence against the human *HOXD9* cDNA, which displayed a marked reduction (69%) in the expression of HOXD9 mRNA in the absence of doxycycline (Figure S4 A). HOXD9 knockdown had no significant effect on the ability of RA to induce morphologic differentiation and G1 arrest (Figure S4 B–D), demonstrating that HOXD9 is not required for RA action, at least in BE(2)-C neuroblastoma cells. These findings also provided the indirect evidence for a specific role of HOXD8 in medicating RA action in BE(2)-C cells.

### HOXC9 is a downstream target of HOXD8 in the RA signaling pathway

**Figure 6:**
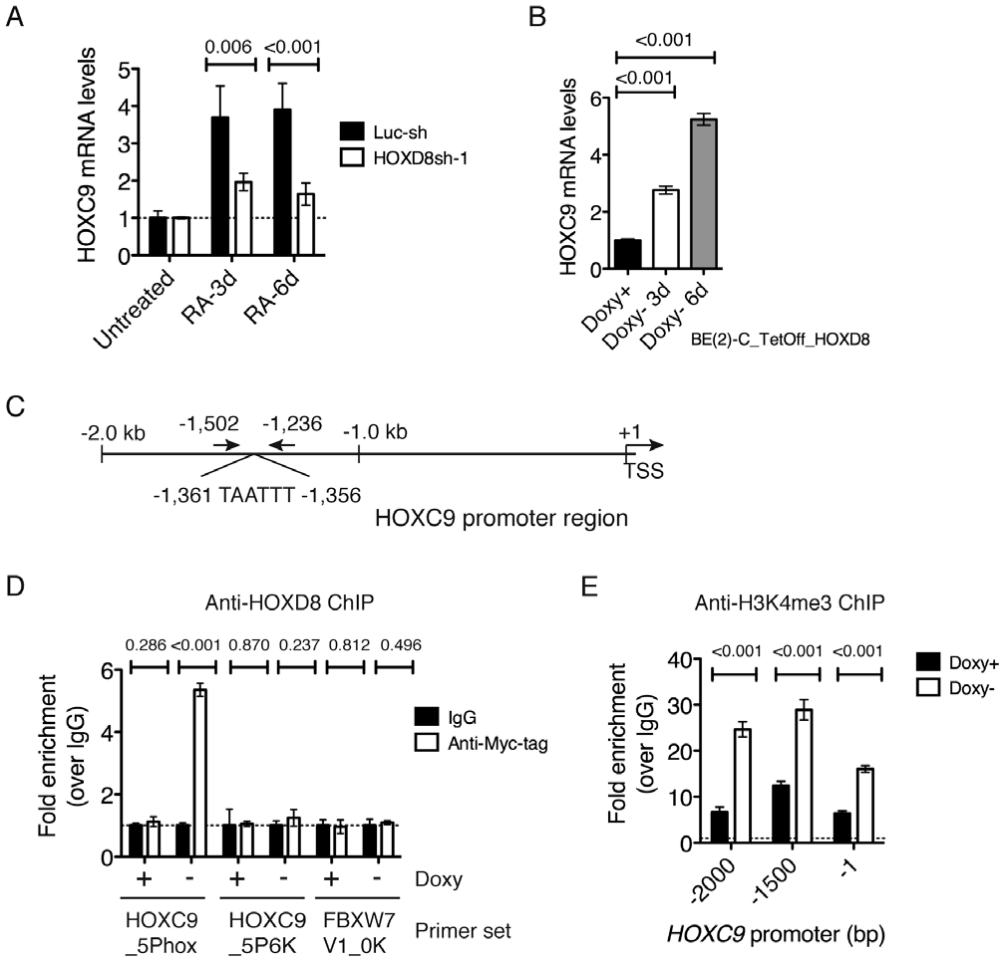
*HOXC9* is a direct target gene of HOXD8. (**A**) qRT-PCR analysis of *HOXC9* mRNA levels in BE(2)-C/Tet-Off/Luc-sh and BE(2)-C/Tet-Off/HOXD8sh-1 cells that were cultured in the absence of Doxy for 6 days and then either untreated or treated with 10 µM RA for 3 or 6 days. The *HOXC9* mRNA levels in BE(2)-C/Tet-Off/Luc-sh cells cultured in the absence of Doxy without RA treatment were designated as 1.0 (dashed line). (**B**) qRT-PCR analysis of *HOXC9* mRNA levels in BE(2)-C/Tet-Off/myc-HOXD8 cells cultured in the presence or absence of Doxy for 3 or 6 days. The *HOXC9* mRNA levels in BE(2)-C/Tet-Off/myc-HOXD8 cells in the presence of Doxy were designated as 1.0. (**C**) Schematic representation of the *HOXC9* promoter region with a potential HOX-binding site 1,361 bp upstream of the transcription start site (TSS,+1). (**D**) ChIP-qPCR analysis of HOXD8 interaction with the *HOXC9* promoter region (-1,502 to -1,236, HOXC9_5Phox) in BE(2)-C/Tet-Off/myc-HOXD8 cells cultured in the presence or absence of Doxy for 6 days, presented as fold enrichment over IgG control (1.0, dashed line). HOXD8 did not bind to the region 6-kb upstream of the *HOXC9* promoter (HOXC9_5P6K) or the TSS of the *FBXW7* gene (FBXW7V1_0K), which is shown as negative control. (**E**) ChIP-qPCR analysis of H3K4me3 levels at the *HOXC9* promoter in BE(2)-C/Tet-Off/myc-HOXD8 cells cultured in the presence or absence of Doxy for 6 days, presented as fold enrichment over the IgG control (1.0, dashed line). The primer pairs target the *HOXC9* promoter region around −2,000, −1,500, and −1 bp. The data (**A**, **B**, **D**, and **E**) were from two independent samples with each being assayed in triplicate and analyzed using two-tailed Student's *t*-test with the *p* values indicated. Error bars, SD.

The above finding that HOXD8 knockdown significantly inhibited the ability of RA to induce differentiation of BE(2)-C cells prompted us to investigate the connection between HOXD8 and HOXC9, which is also critical for RA action in neuroblastoma cells [25]. HOXD8 knockdown significantly inhibited RA-induced HOXC9 expression (Figure 6 A). In addition, induction of HOXD8 alone was sufficient to upregulate HOXC9 expression (Figure 6 B). We further investigated the possibility of *HOXC9* as a direct target gene of HOXD8. HOX proteins bind DNA sequences with a conserved TAATT/AA-motif [3], [37]. Sequence examination revealed multiple potential HOX-binding sites within the 2-kb 5′ flanking region of the human *HOXC9* gene, with one matching closely to the consensus sequence located −1,361 bp upstream of the transcription start site (TSS,+1; Figure 6 C). ChIP-qPCR analysis revealed significant interaction of HOXD8 with the *HOXC9* promoter region spanning from−1,502 to−1,236 bp (5.4-fold enrichment relative to IgG control, HOXC9_5Phox, Figure 6 D). By contrast, we observed no detectable association of HOXD8 with the region 6-kb upstream of the *HOXC9* TSS (HOXC9_5P6K) or with the TSS of the ubiquitin ligase gene *FBXW7* (FBXW7V1_0K, Figure 6 D). Together, these findings demonstrate that HOXD8 directly and specifically binds to the *HOXC9* promoter-proximal region spanning from−1,502 to−1,236 bp in the context of chromatin.

We next examined the chromatin state of the *HOXC9* promoter by ChIP-qPCR using an antibody against trimethylated histone H3 at lysine 4 (H3K4me3), which marks a transcriptionally active state [38]. Consistent with the observed transcriptional activation of *HOXC9*, ChIP-qPCR assays revealed a significant increase in the levels of H3K4me3 (2.4–3.7 fold over the basal levels) at the *HOXC9* promoter following *HOXD8* induction (Figure 6 E). Together, these findings indicate that HOXD8 directly targets *HOXC9* for transcriptional activation.

## Discussion

In this report, we present three major findings from our comprehensive study of all *HOXD* genes for their functions in the control of neuroblastoma cell proliferation and differentiation in response to RA. First, all *HOXD* genes are induced by RA, which displays an expression pattern similar to what was originally observed in human embryonal carcinoma cells: *HOX* genes located at the 3′ end are activated generally earlier by RA than those positioned more 5′ within the cluster [11], [12]. Second, several human HOXD proteins corresponding to the *Drosophila* Hox proteins abd-A (HOXD8) and Abd-B (HOXD9, HOXD10, HOX12) are functionally equivalent in promoting cell cycle arrest and neuronal differentiation of BE(2)-C cells, with the ability to recapitulate the phenotype of RA action. Finally, HOXD8 is a mediator of RA action in neuroblastoma cells by transcriptional activation of the *HOXC9* gene, suggesting an RA-HOXD8-HOXC9 pathway in driving neuroblastoma cell differentiation. These findings have implications both in developing differentiation-based therapies for neuroblastoma and in employing neuroblastoma cells as a model system for investigating the molecular basis of *HOX* genes in regulation of neuronal differentiation.

RA has been used in clinic as a differentiation agent for treatment of high-risk neuroblastomas [23], [24]. In combination with bone-marrow transplant, RA treatment can significantly improve the event-free survival of high-risk neuroblastoma patients [39], [40]. However, resistance to RA presents a major barrier to successful RA-based therapy [23], [24]. Identification of downstream genes that are mediators of RA-induced differentiation may offer opportunities to bypass resistance to RA. In this study, we show that individual induction of *HOXD8, HOXD9, HOXD10* or *HOXD12* in neuroblastoma cells is able to recapitulate RA-induced differentiation phenotype. Moreover, we identify HOXD8 as a key component of the RA signaling pathway. We show that RA induction of *HOXD8* is critical for the differentiation process at both the cellular and molecular levels: Downregulation of HOXD8 expression interferes with RA-induced morphologic differentiation and G1 arrest, as well as upregulation of neuronal genes and downregulation of cell cycle genes. We further present evidence that *HOXC9*, also a key mediator of RA action in neuroblastoma cells [25], is a direct target gene of HOXD8. Importantly, HOXD8 knockdown significantly inhibits the ability of RA to upregulate HOXC9 expression, providing both the physiological relevance and a molecular mechanism to the function of HOXD8 in the RA signaling pathway. Interestingly, HOXD9, despite its ability to induce neuronal differentiation and G1 arrest, is not essential for RA action, at least in BE(2)-C cells. The roles of HOXD10 and HOXD12 in the RA signaling pathway remain to be investigated. Nonetheless, our finding that induction of many *HOXD* genes can fully recapitulate RA action in neuroblastoma cells suggests that pharmacological activation of *HOXD* cluster genes may represent a therapeutic strategy for RA-resistant neuroblastomas.

Early studies in the human embryonal carcinoma cell line NT2/D1 have shown that RA differentially activates *HOX* genes according to their location within the four (*HOXA-D*) clusters, in a time- and RA concentration-dependent fashion. This order corresponds to the expression patterns in developing axial systems in the human and mouse where 3′ genes are expressed earlier and 5′ genes later [28], [29]. These findings indicate that the temporal response of *HOX* genes to RA in human embryonal carcinoma cells is of physiological relevance, leading to the suggestion that a molecular understanding of the RA response in cell-based systems may provide important insights into the temporal regulation of *HOX* gene expression in the embryo [41]. In NT2/D1 cells, RA induces all *HOXB* genes as well as 3′ *HOXA, HOXC* and *HOXD* genes roughly corresponding to the *HOX-5* paralog groups [11], [12]. This is in striking contrast to BE(2)-C cells in which RA induces all *HOXD* genes, which largely recapitulates the sequential activation of *Hoxd* genes observed in the mouse embryo [42]. Thus, RA induction of *HOXD* genes in neuroblastoma cells represents a valuable model for studying the molecular mechanism that controls the temporal expression of *HOXD* genes.

Studies with *Drosophila Hox* genes have provided convincing evidence that each *Hox* gene specifies a unique morphology along the anteroposterior axis. Therefore, replacing one *Hox* gene with another will switch the identity of the tissue in which it is expressed during development, a phenomenon called homeotic transformation [43]. However, in mammals, there is evidence that some *Hox* genes are interchangeable and redundant. For example, *Hoxa3^−/−^* mice die at or immediately after birth [44], which can be fully rescued by replacing the protein-coding sequence of *Hoxa3* with that of *Hoxd3* [45]. Also, replacing the homeodomain of *Hoxa11* with that of *Hoxa10* in mice can provide wild-type function for the missing *Hoxa11* gene in the development of the axial skeleton and male reproduction track [46]. Our study has uncovered the same principle in a cell-based system. HOXD8, HOXD9, HOXD10 and HOXD12 proteins are functionally equivalent in inducing cell cycle arrest and neuronal differentiation of BE(2)-C cells. Also interestingly, other HOXD proteins are functionally distinct, even within the same cellular context: HOXD1 has no phenotypic effect; HOXD3 is able to induce cell cycle arrest and morphologic differentiation but fails to upregulate the neuronal marker NEFM; HOXD4, HOXD11 and HOXD13 are able to upregulate NEFM but cannot induce cell cycle arrest and morphologic differentiation.

It is important to point out that in addition to the temporal regulation, the expression of *HOX* genes is spatially regulated, showing a direct correlation between their linear arrangement along the chromosomes and the anteroposterior boundaries of their expression [47]. This spatial colinearity is critical for the developmental function of *HOX* genes in specifying positional identities of tissues along the anteroposterior axis. Notably, the four human HOXD proteins (HOXD8, HOXD9, HOXD10, HOXD12) with both anti-growth and differentiation-inducing activities in neuroblastoma cells correspond to the *Drosophila* Hox proteins abd-A (HOXD8) and Abd-B (HOXD9, HOXD10, HOX12) that are responsible for specifying the abdomen [43]. Most of neuroblastomas occur in the adrenal gland and abdomen [16]. Thus, we suspect that BE(2)-C neuroblastoma cells may provide a specific cellular environment (e.g., co-factors) necessary for the four HOXD proteins to exert their functions. Together, these observations make neuroblastoma cells an attractive system for uncovering the molecular mechanisms that govern the functions of HOXD proteins in regulation of neuroblastoma cell proliferation and neuronal differentiation.

## Materials and Methods

### Cell culture and inducible expression of *HOXD* genes

The human neuroblastoma cell line BE(2)-C (CRL-2268, ATCC) was cultured in a 1∶1 mixture of DMEM and Ham's nutrient mixture F12 supplemented with 10% fetal bovine serum (Invitrogen). The Retro-X Tet-Off Advanced Inducible Gene Expression System (Clontech) was used to generate BE(2)-C-derived cell lines with inducible expression of individual *HOXD* genes in the absence of doxycycline. Myc-tagged human *HOXD* genes were generated by PCR, verified by sequencing, and subcloned into pRetroX-Tight-pur. cDNAs coding for individual HOXD proteins were obtained from Open Biosystems, OriGene, ATCC, Addgene, and Invitrogen. Retroviruses were produced in 293FT cells using the packaging plasmids pHDM-G and pMD.MLVogp. For morphologic examination of neuronal differentiation, cells were examined and phase contrast images captured daily using an Axio Observer microscope and the software AxioVision (Carl Zeiss MicroImaging).

### RA treatment

*All trans*-RA (Sigma-Aldrich) was dissolved in DMSO and 10 mM stock solutions were prepared. BE(2)-C cells were treated with 10 µM RA. DMSO at the final concentration of 0.1%was used as negative control (untreated). Cells were examined and phase contrast images captured using an Axio Observer microscope and the software AxioVision.

### Fluorescence-activated cell sorting (FACS)

Cells were fixed in 70%ethanol, incubated with ribonuclease A (Sigma-Aldrich), and stained with 20 mg/ml propidium iodide (Invitogen). Samples were analyzed with a FACSCalibur system and ModFitLT V3.2.1 software (BD Bioscience).

### Quantitative reverse-transcription PCR (qRT-PCR)

Total RNA was purified from cells using Trizol (Invitrogen). Reverse transcriptions were performed using SuperScript II Reverse Transcriptase (Invitrogen). Quantitative real-time PCR were performed using a RT^2^ SYBR green/Fluorescein PCR master mix (SABiosciences). The primer sequences for *HOXD* genes have been described previously [48] and for *CCNB1, CDK1*, *HOXC9, NEFM, RET*, and *GAPDH* are listed in Table S1. All primer pairs were verified by melting curve analysis following qRT-PCR, with each primer pair showing a single desired amplification peak. Samples from at least two independent experiments were analyzed by qRT-PCR and each sample was assayed in triplicate. PCR was performed on an iQ5 real-time PCR system (Bio-Rad) and the data from two or more independent samples were combined and analyzed using the GFX Manager Gene Expression Analysis software (Bio-RAD).

### Immunoblotting

Cells were suspended in standard SDS sample buffer and protein concentrations were determined using a protein assay kit (Bio-Rad) with bovine serum albumin as reference. Proteins (50 µg) were separated on SDS-polyacrylamide gels, transferred to nitrocellulose membranes, and probed with the following primary antibodies: mouse anti-Myc-tag (9E10, hybridoma supernatant, 1∶10), rabbit anti-cyclin A2 (sc-751, 1∶200), mouse anti-cyclin B1 (sc-245, 1∶200), mouse anti-cyclin D1 (sc-20044, 1∶200), mouse anti-CDK1 (610037, 1∶1000; BD Biosciences), rabbit anti-CDK4 (sc-260, 1∶200), rabbit anti-CDK6 (sc-177, 1∶200), rabbit anti-PHOX2B (1∶1000) [49], mouse anti-NEFM (NF-09, sc-51683, 1∶200), mouse anti-alpha-tubulin (B-5-1-2, 1∶5000; Sigma-Aldrich), and rabbit anti-beta-actin (600-401-886, 1∶2000; Rockland Immunochemicals). Unless indicated, all primary antibodies were purchased from Santa Cruz Biotechnology. For detecting NEFM, PHOX2B, cyclin A2, cyclin D1, CDK4, CDK6, Myc-tag, alpha-tubulin and beta-actin, membranes were incubated with IRDye-labeled secondary antibodies and scanned on an Odyssey Infrared Imaging System (LI-COR Biosciences). For detecting cyclin B1 and CDK1, membranes were incubated with Horseradish peroxidase conjugated goat anti-mouse IgG (1∶5000, Santa Cruz Biotechnology). Proteins were visualized using a SuperSignal West Pico chemiluminescence kit (Pierce).

### Immunofluorescence

Cells were fixed with 4% paraformaldehyde and stained with rabbit anti-KI67 (sc-15402, 1∶100) and mouse anti-NEFM (NF-09, sc-51683, 1∶100). All secondary fluorescence antibodies were from Molecular Probes and used at 1∶800 dilutions for goat anti-mouse (Alexa Fluor 488) and goat anti-rabbit (Alexa Fluor594). Nuclei were stained with DAPI. Fluorescent images were captured with a fluorescence microscope (Carl Zeiss Axio Observer).

### RNA interference

Retroviral (pSM2) constructs expressing shRNA to human *HOXD8* (RHS1764-9501133) or *HOXD9* (RHS1764-9691903) were purchased from Open Biosystems and the shRNA-coding sequences were subcloned into the retroviral shRNAmir Tet-Off vector [50] for inducible expression in BE(2C)-C/Tet-Off cells in the absence of doxycycline [25]. The luciferase shRNAmir Tet-Off construct was used as control. Retroviruses were produced in 293FT cells using the packaging plasmids pHDM-G and pMD.MLVogp.

### Chromatin Immunoprecipitation (ChIP)

ChIP assays were performed on BE(2)-C/Tet-Off/myc-HOXD8 cells cultured in the presence (2 µg/ml) or absence of doxycycline for 3 days, using mouse anti-Myc tag (clone 4A6, Millipore), rabbit anti-H3K4me3 (07–473, Millipore), or control mouse or rabbit IgG according to the online protocol (http://jura.wi.mit.edu/young_public/hESregulation/ChIP.html). Two independent ChIP genomic DNA samples were analyzed and each sample was assayed in triplicate by qPCR. Primer sequences for ChIP-qPCR assays are listed in Table S2.

### Quantification and statistical analysis

For KI67 immunofluorescence staining, approximately 400 cells (DAPI-positive) were counted from at least 5 randomly selected 400x fields independently by three investigators, and the percentage of KI67^+^ cells was determined. All quantitative data including cell cycle analysis, KI67 staining, and qRT-PCR were analyzed between test and control groups using two-tailed Student's *t*-test, and *p* value <0.05 was considered to be statistically significant.

## Supporting Information

Figure S1 Generation of BE(2)-C-derived cell lines with inducible expression of individual *HOXD* genes. (**A**) qRT-PCR analysis of *HOXD* mRNA levels in BE(2)-C/Tet-Off/myc-HOXD cells cultured in presence (Doxy+, 2 µg/ml) or absence of doxycycline (Doxy-) for 3 days. The *HOXD* mRNA levels in the presence of Doxy were designated as 1.0. The data were from two independent samples with each being assayed in triplicates. Error bars, SD. (**B**) Immunoblot analysis of myc-HOXD proteins in the absence of Doxy for 3 or 6 days. Beta-actin levels are shown as loading control.
(TIF)

Figure S2 Induction of HOXD genes has no effect on the expression of cyclin D1, CDK4 and CDK6. Immunoblot analysis of BE(2)-C/Tet-Off/myc-HOXD cells cultured in the presence (2 µg/ml) or absence of Doxy for 3 or 6 days (the same cell samples shown in Figure 3). Alpha-tubulin levels are shown as loading control. The data are representative of two independent experiments with similar results.
(TIF)

Figure S3 HOXD8 is a mediator of RA action. (**A**) qRT-PCR analysis of HOXD8 mRNA levels in BE(2)-C/Tet-Off/Luc-sh and BE(2)-C/Tet-Off/HOXD8sh-2 cells in the presence (2 µg/ml) or absence of Doxy for 6 days. The HOXD8 mRNA level in BE(2)-C/Tet-Off/luc-sh cells in the presence of Doxy was designated as 1.0. The induction of HOXD8sh-2 resulted in an average of 68% reduction in HOXD8 mRNA levels. The data were from two independent samples with each being assayed in triplicate and analyzed using two-tailed Student's *t*-test with the *p* values indicated. Error bars, SD. (**B**) FACS analysis of the cell cycle status of BE(2)-C/Tet-Off/Luc-sh and BE(2)-C/Tet-Off/HOXD8sh-2 cells that were cultured in the absence of Doxy for 6 days and then either untreated or treated with 10 µM RA for 3 or 6 days. Shown are representatives of two independent experiments with similar results.
(TIF)

Figure S4 HOXD9 is not required for RA action. (**A**) qRT-PCR analysis of HOXD9 mRNA levels in BE(2)-C/Tet-Off/Luc-sh and BE(2)-C/Tet-Off/HOXD9-sh cells cultured in the presence (2 µg/ml) or absence of Doxy for 6 days. The HOXD9 mRNA level in BE(2)-C/Tet-Off/Luc-sh cells in the presence of Doxy was designated as 1.0. The induction of HOXD9-sh resulted in an average of 69% reduction in HOXD9 mRNA levels. The data were from two independent samples with each being assayed in triplicate and analyzed using two-tailed Student's *t*-test with the *p* values indicated. (**B–D**) Phase contrast imaging (**B**) and cell cycle analysis (**C–D**) of BE(2)-C/Tet-Off/Luc-sh and BE(2)-C/Tet-Off/HOXD9-sh cells that were cultured in the absence of Doxy for 6 days and then either untreated or treated with 10 µM RA for 3 or 6 days. The data presented in (**D**) were from three independent experiments and analyzed using two-tailed Student's *t*-test with the *p* values indicated. Error bars (**A** and **D**), SD.
(TIF)

Table S1 qRT-PCR primers.
(DOC)

Table S2 ChIP-qPCR primers.
(DOC)
