# Supplementary figures and images for: Functional Dissection of HOXD Cluster Genes in Regulation of Neuroblastoma Cell Proliferation and Differentiation
Source: PLoS One. 2012 Aug 7;7(8):e40728. doi: 10.1371/journal.pone.0040728 (PMC3413684; doi:10.1371/journal.pone.0040728)

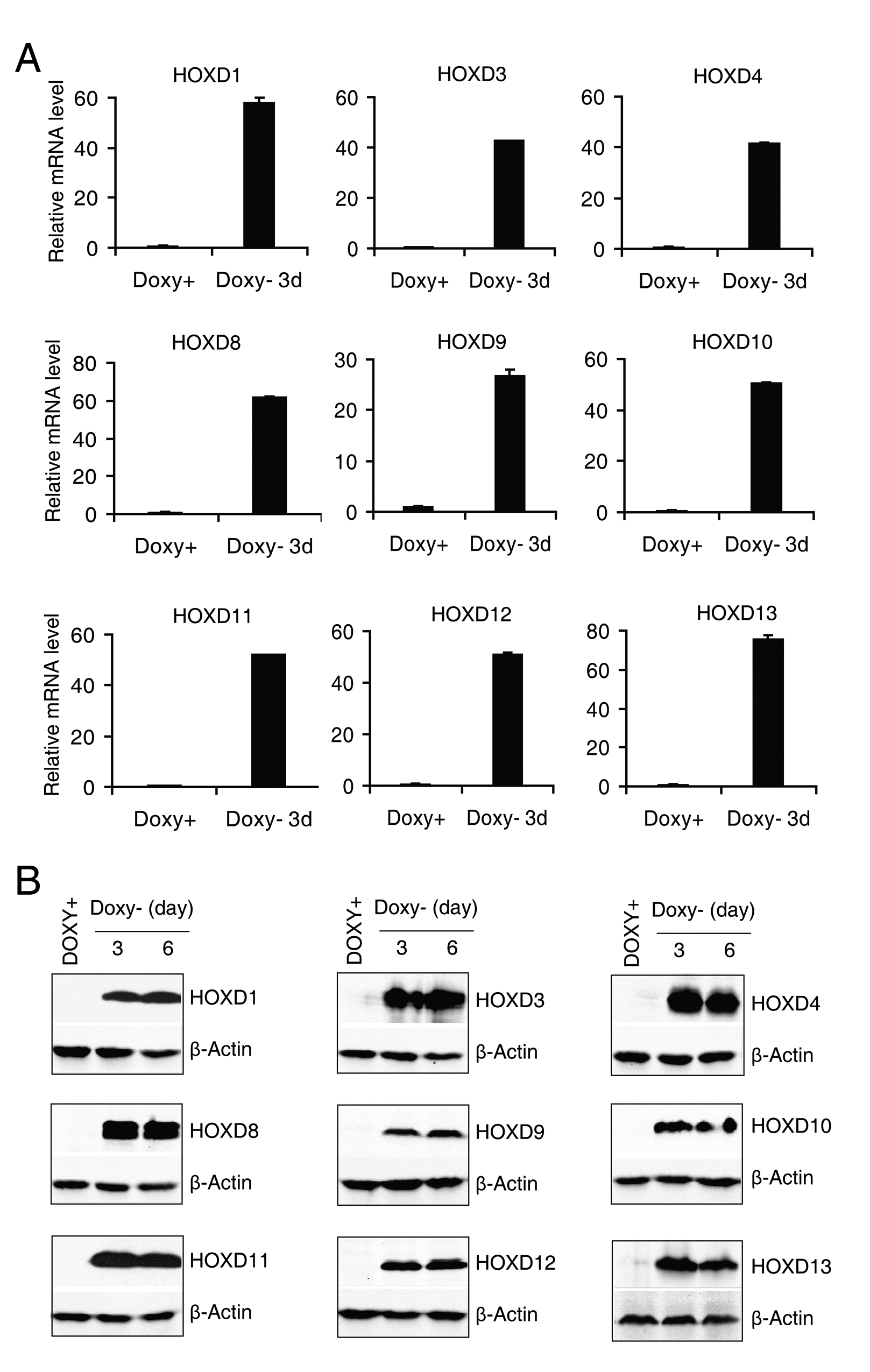

Supplement: Figure S1 — Generation of BE(2)-C-derived cell lines with inducible expression of individual HOXD genes. (A) qRT-PCR analysis of HOXD mRNA levels in BE(2)-C/Tet-Off/myc-HOXD cells cultured in presence (Doxy+, 2 µg/ml) or absence of doxycycline (Doxy-) for 3 days. The HOXD mRNA levels in the presence of Doxy were designated as 1.0. The data were from two independent samples with each being assayed in triplicates. Error bars, SD. (B) Immunoblot analysis of myc-HOXD proteins in the absence of Doxy for 3 or 6 days. Beta-actin levels are shown as loading control. (TIF) [file pone.0040728.s001.tif]

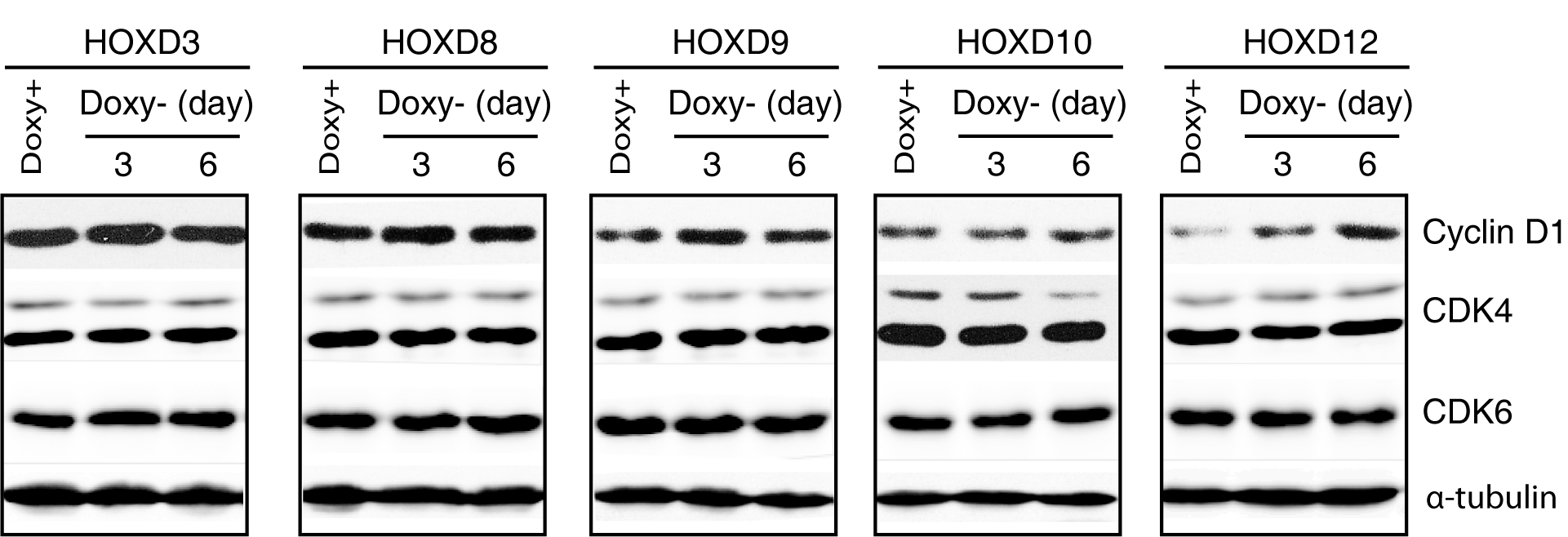

Supplement: Figure S2 — Induction of HOXD genes has no effect on the expression of cyclin D1, CDK4 and CDK6. Immunoblot analysis of BE(2)-C/Tet-Off/myc-HOXD cells cultured in the presence (2 µg/ml) or absence of Doxy for 3 or 6 days (the same cell samples shown in Figure 3). Alpha-tubulin levels are shown as loading control. The data are representative of two independent experiments with similar results. (TIF) [file pone.0040728.s002.tif]

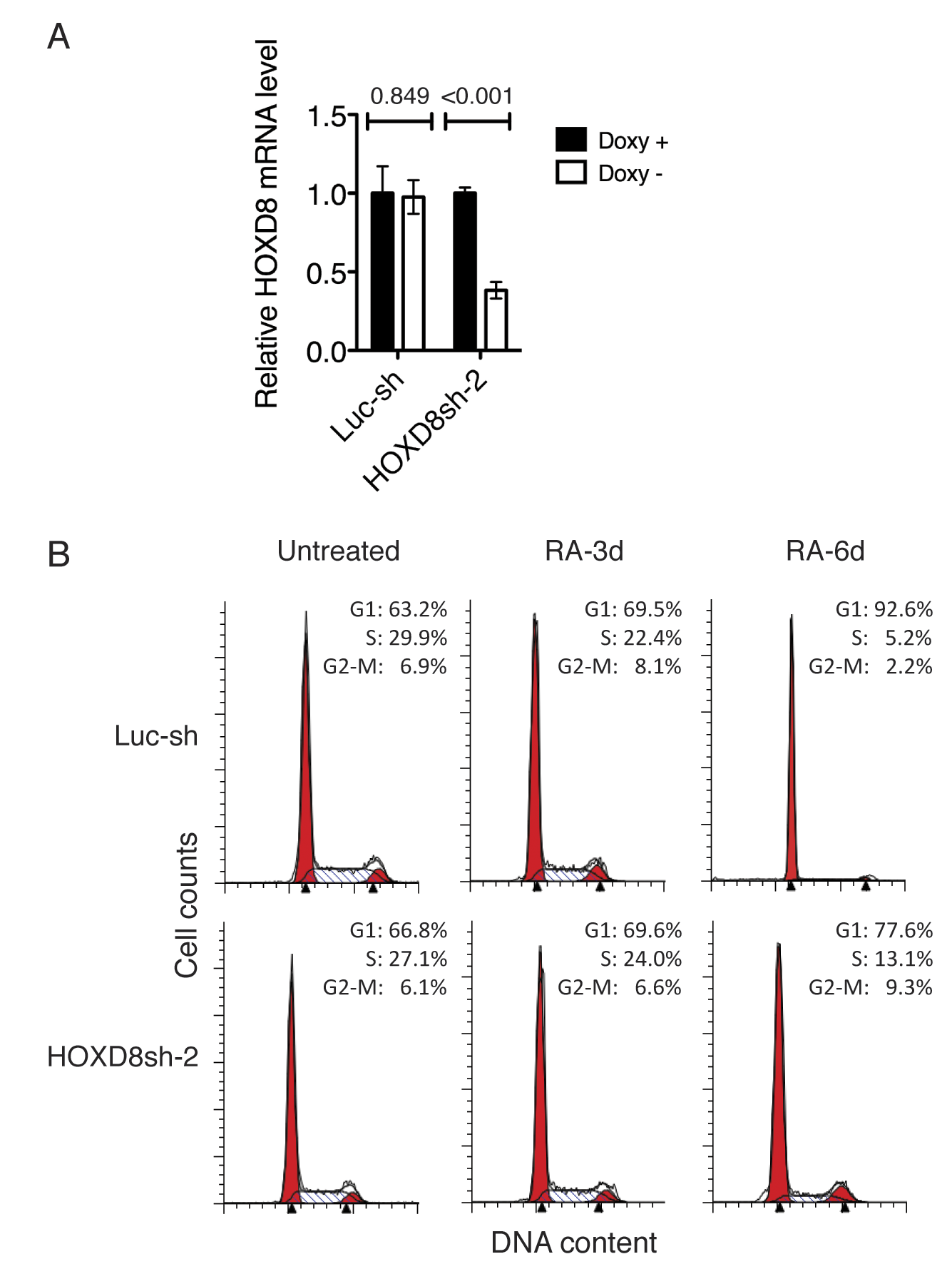

Supplement: Figure S3 — HOXD8 is a mediator of RA action. (A) qRT-PCR analysis of HOXD8 mRNA levels in BE(2)-C/Tet-Off/Luc-sh and BE(2)-C/Tet-Off/HOXD8sh-2 cells in the presence (2 µg/ml) or absence of Doxy for 6 days. The HOXD8 mRNA level in BE(2)-C/Tet-Off/luc-sh cells in the presence of Doxy was designated as 1.0. The induction of HOXD8sh-2 resulted in an average of 68% reduction in HOXD8 mRNA levels. The data were from two independent samples with each being assayed in triplicate and analyzed using two-tailed Student's t-test with the p values indicated. Error bars, SD. (B) FACS analysis of the cell cycle status of BE(2)-C/Tet-Off/Luc-sh and BE(2)-C/Tet-Off/HOXD8sh-2 cells that were cultured in the absence of Doxy for 6 days and then either untreated or treated with 10 µM RA for 3 or 6 days. Shown are representatives of two independent experiments with similar results. (TIF) [file pone.0040728.s003.tif]

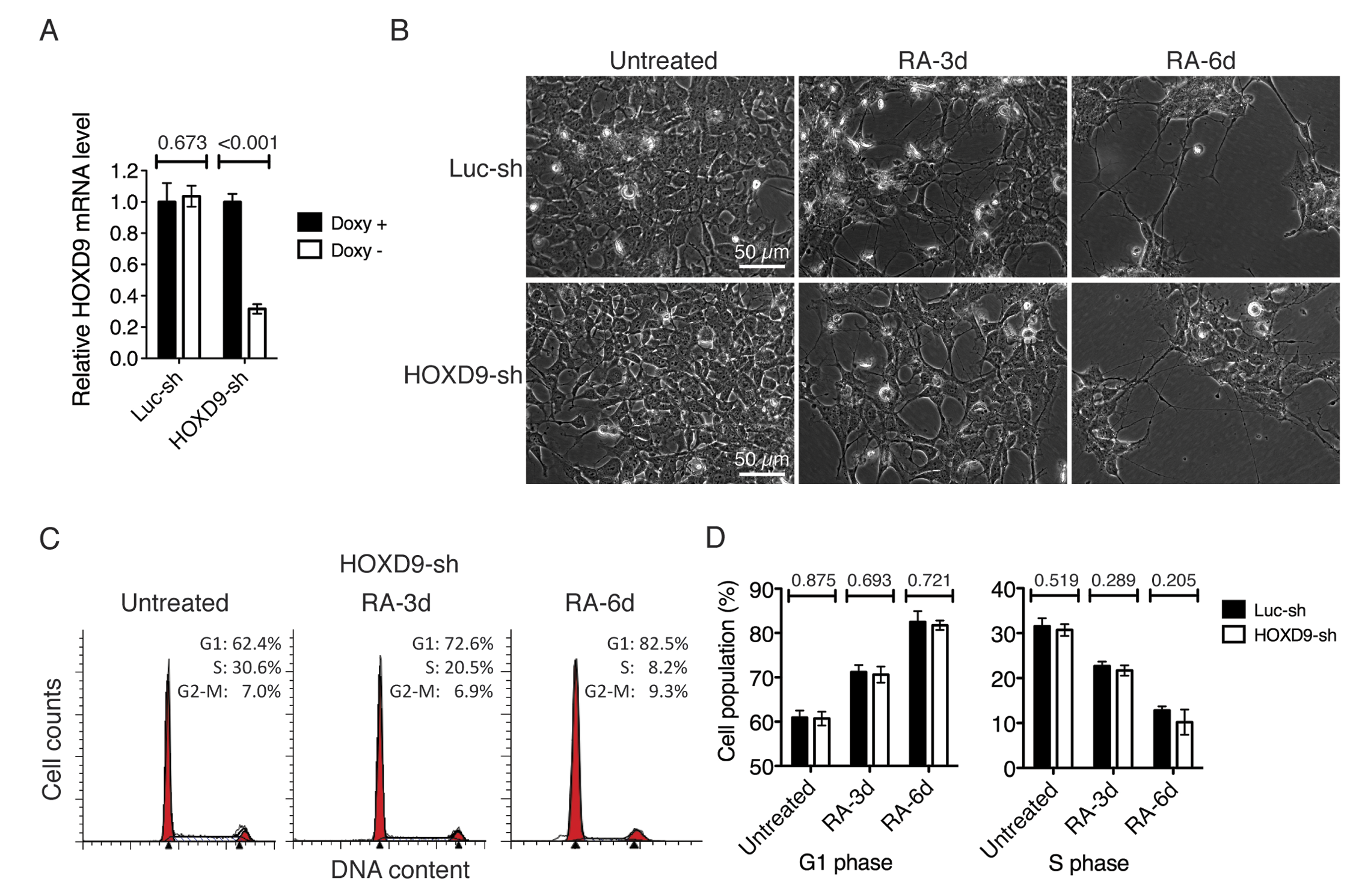

Supplement: Figure S4 — HOXD9 is not required for RA action. (A) qRT-PCR analysis of HOXD9 mRNA levels in BE(2)-C/Tet-Off/Luc-sh and BE(2)-C/Tet-Off/HOXD9-sh cells cultured in the presence (2 µg/ml) or absence of Doxy for 6 days. The HOXD9 mRNA level in BE(2)-C/Tet-Off/Luc-sh cells in the presence of Doxy was designated as 1.0. The induction of HOXD9-sh resulted in an average of 69% reduction in HOXD9 mRNA levels. The data were from two independent samples with each being assayed in triplicate and analyzed using two-tailed Student's t-test with the p values indicated. (B–D) Phase contrast imaging (B) and cell cycle analysis (C–D) of BE(2)-C/Tet-Off/Luc-sh and BE(2)-C/Tet-Off/HOXD9-sh cells that were cultured in the absence of Doxy for 6 days and then either untreated or treated with 10 µM RA for 3 or 6 days. The data presented in (D) were from three independent experiments and analyzed using two-tailed Student's t-test with the p values indicated. Error bars (A and D), SD. (TIF) [file pone.0040728.s004.tif]
