# Supplementary material for: Functional Dissection of HOXD Cluster Genes in Regulation of Neuroblastoma Cell Proliferation and Differentiation
Source: PLoS One. 2012 Aug 7;7(8):e40728. doi: 10.1371/journal.pone.0040728 (PMC3413684; doi:10.1371/journal.pone.0040728)
Supplement: Table S1 — qRT-PCR primers. (DOC) [file pone.0040728.s005.doc]

| Table S1. qRT-PCR primers | | |
| --- | --- | --- |
| Primer set | Forward (5'-3') | Reverse (5'-3') |
| CCNB1 | GACCTGTGTCAGGCTTTCTCTG | GGTATTTTGGTCTGACTGCTTGC |
| CDK1 | TGATCCAGCCAAACGAATTTC | GCTACATCTTCTTAATCTGATTGTCCAA |
| HOXC9 | CTCGCTCATCTCTCACGACAA | GACGGAAAATCGCTACAGTCC |
| NEFM | AGTGGTTCAAATGCCGCTAC | TTTTCCAACTGCTGGATGGT |
| RET | GGCATCAACGTCCAGTACAAG | TGAGGTGACCACCCCTAGC |
| GAPDH | GAGTCAACGGATTTGGTCGT | TTGATTTTGGAGGGATCTCG |
