# Supplementary material for: Functional Dissection of HOXD Cluster Genes in Regulation of Neuroblastoma Cell Proliferation and Differentiation
Source: PLoS One. 2012 Aug 7;7(8):e40728. doi: 10.1371/journal.pone.0040728 (PMC3413684; doi:10.1371/journal.pone.0040728)
Supplement: Table S2 — ChIP-qPCR primers. (DOC) [file pone.0040728.s006.doc]

| Table S2. ChIP-qPCR primers | | |
| --- | --- | --- |
| Primer set | Forward (5'-3') | Reverse (5'-3') |
| HOXC9_5P0K | CGCATTGATCCGCGCCGTATT | AGAGATGAGCGAGTCCACGTA |
| HOXC9_5P1.5K | GAGCCTGCATCTCAACCTCAG | CCAGAAATATGGTGCACTTAG |
| HOXC9_5P2K | GGACTGGAACCTCCTGTCTTT | CTCCCTTGAGCTGGACTGAGC |
| HOXC9_5P6K | TGAGATCTCTCGTCGGCTGCT | TCCGAGCCGAAGTCAGGCCGT |
| HOXC9_5Phox | CCTTTCTGACCCTGAAATTGC | AATATCAGACAACTTGGATTC |
| FBXW7V1_0K | AGGAAAAGAGGAGG AAGCG | TCGTGTCGCTAAACCAGGCGG |
